# Targeting protein arginine methyltransferase 5 inhibits colorectal cancer growth by decreasing arginine methylation of eIF4E and FGFR3

**DOI:** 10.18632/oncotarget.4332

**Published:** 2015-06-02

**Authors:** Baolai Zhang, Shuhong Dong, Ruiming Zhu, Chunyan Hu, Jing Hou, Yan Li, Qian Zhao, Xue Shao, Qian Bu, Hongyu Li, Yongjie Wu, Xiaobo Cen, Yinglan Zhao

**Affiliations:** ^1^ State Key Laboratory of Biotherapy and Cancer Center, West China Hospital, Sichuan University, and Collaborative Innovation Center for Biotherapy, Chengdu, China; ^2^ Department of Pharmacology, School of Basic Medical Sciences, Lanzhou University, Lanzhou, China

**Keywords:** PRMT5, AMI-1, colorectal cancer, FGFR3, eIF4E

## Abstract

Protein arginine methyltransferases (PRMTs) plays critical roles in cancer. PRMT5 has been implicated in several types of tumors. However, the role of PRMT5 in cancer development remains to be fully elucidated. Here, we provide evidence that PRMT5 is overexpressed in colorectal cancer (CRC) cells and patient-derived primary tumors, correlated with increased cell growth and decreased overall patient survival. Arginine methyltransferase inhibitor 1 (AMI-1)strongly inhibited tumor growth, increased the ratio of Bax/Bcl-2, and induced apoptosis in mouse CRC xenograt model. AMI-1 also induced apoptosis and decreased the migratory activity in several CRC cells. In CRC xenografts AMI-1 significantly decreased symmetric dimethylation of histone 4 (H4R3me2s), a histone mark of type II PRMT5, but not the expression of H4R3me2a, a histone mark of type I PRMTs. These results suggest that the inhibition of PRMT5 contributes to the antitumor efficacy of AMI-1. Chromatin immunoprecipitation (ChIP) identified FGFR3 and eIF4E as two key genes regulated by PRMT5. PRMT5 knockdown reduced the levels of H4R3me2s and H3R8me2s methylation on FGFR3 and eIF4E promoters, leading to decreased expressions of FGFR3 and eIF4E. Collectively, our findings provide new evidence that PRMT5 plays an important role in CRC pathogenesis through epigenetically regulating arginine methylation of oncogenes such as eIF4E and FGFR3.

## INTRODUCTION

Colorectal cancer (CRC) is the third most common cancer and the fourth most common cancer cause of death globally [1]. Most cases of CRC are sporadic and develop slowly over more than 10 years through the adenoma-carcinoma sequence [2]. *APC* gene mutation is an early event in the multistep process of CRC and occurs in more than 70% of colorectal adenoma. The adenoma-carcinoma sequence is further promoted by activating mutations of *KRAS* oncogene and inactivating mutations of *TP53* tumor suppressor gene [3]. However, more than 15% of sporadic CRC develop through fundamentally different pathways. These cancers include those originating from serrated precursor lesions, and are often characterized by CpG island methylation and activating mutations of *BRAF* oncogene [4]. Nevertheless, molecular pathogenesis of CRC is heterogeneous and poorly understood.

Arginine methylation is crucially involved in the regulation of gene expression, RNA metabolism, cell cycle, and protein function [5-7]. Protein arginine methyltransferases (PRMTs) use *S*-adenosylmethionine (AdoMet) as methyl donor to catalyze the transfer of methyl groups to the arginine residues of histones H3 and H4 or non-histones [6, 8]. The type I PRMTs (PRMT1, 3, 4, 6 and 8) in mammalian cells specifically deposit an asymmetric dimethylarginine (ADMA) mark on histone 4 at arginine 3 (H4R3me2a), whereas type II PRMTs (PRMT5) catalyze the formation of symmetric dimethylarginine [5, 9]. PRMT5-driven methylation of arginine residues leads to symmetric dimethylation of histone H3 (H3R8me2s) and H4 (H4R3me2s), which in turn alters chromatin structure to promote transcriptional repression [10-12]. Indeed, PRMT5 has been found to be overexpressed in leukemia, lymphoma, lung cancer and breast cancer [6, 13-15]. In a collection of biopsies taken from CRC patients, elevated level of PRMT5 was associated with low expression of E2F1 [16]. PRMT5-directed methylation of tumor suppressor p53 has been shown in cells with DNA damage [17, 18]. PRMTs also methylate the promoters of epidermal growth factor receptors (EGFR) and forkhead box O transcription factors to promote cell survival and growth [19, 20].

In this study, we aimed to characterize the role of PRMT5 in CRC and elucidate the underlying mechanism. Our data show that PRMT5 was overexpressed in approximately 75% of cases. Inhibition of PRMT5 by AMI-1 or knockdown of PRMT5 by siRNA in CRC led to the restoration of critical regulatory pathways affecting cell growth, survival, migration and tumor suppressor activity.

## RESULTS

### PRMT5 expression is frequently increased in human CRC tissues and cells

**Figure 1:**
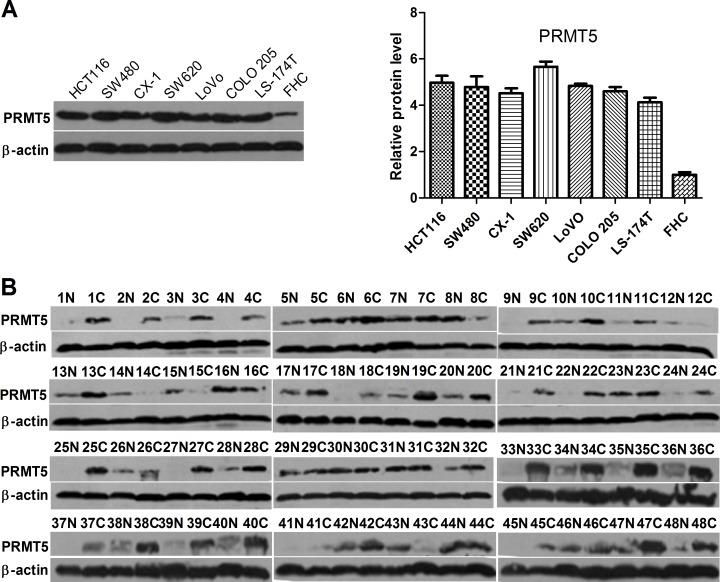
PRMT5 expression in colorectal tumor and cell lines **A.** Western blot analysis of PRMT5 expression in human normal colonic mucosal FHC cells and CRC cell lines. **B.** Western blot analysis of PRMT5 expression in CRC tissues **C.** and NATs (N).

To evaluate the expression levels of PRMT5 in CRC cells, we performed Western blot analysis to determine protein levels of PRMT5 in several CRC cell lines. In comparison with normal colonic mucosal FHC cells, all seven human CRC cell lines expressed high levels of PRMT5 (Figure 1A). To further investigate the expression of PRMT5 in clinical CRC samples, we performed Western blot analysis on a cohort of 48 primary CRC tissues. Of the 48 tumor samples, PRMT5 expression was elevated significantly in 36 (75%) CRC tissues compared to the corresponding normal adjacent tissues (NATs) (Figure 1B).

**Figure 2:**
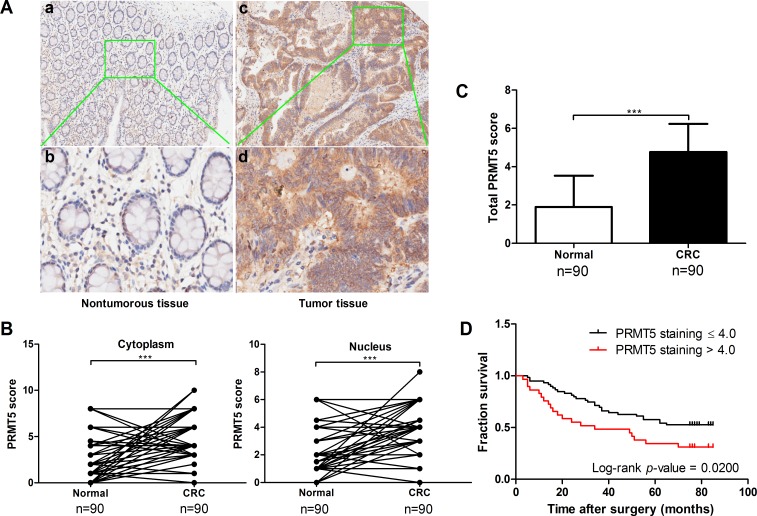
PRMT5 is overexpressed in CRCs and negatively correlated with patient survival **A.** Immunohistochemical staining of PRMT5 in CRC tissues and corresponding NATs. Positive cells were stained brown. Magnification, (a, c) 100×; (b, d,) 400×. **B.** Plot representation of scores based on the nuclear or cytoplasmic expression of PRMT5 in 90 CRC patients compared with matched normal tissues. **C.** Total PRMT5 score in adjacent normal and tumor tissues. **D.** Correlation analysis of PRMT5 expression status in the nucleus and patient survival.

To confirm the clinical significance of PRMT5 overexpression in human CRC development, we analyzed PRMT5 level and its distribution in CRC tissue array by immunohistochemical staining. The array included cancer tissue samples and corresponding NATs from 90 CRC patients (47 males and 43 females, average age 69 years old, range 24-90 years). Among these patients, 46 (51.1%) died of tumor related causes and 44 (48.9%) were still alive at the last follow-up (Table 1). PRMT5 levels in the tissues array specimens were assessed by staining density scores. We found that in the cancer tissues, 56 cases (62.2%) exhibited strong immunopositivity, 30 cases (33.3%) exhibited moderate immunopositivity, and 4 cases (4.4%) exhibited no or weak immunopositivity. In contrast, the majority of normal tissues (70.0%) exhibited no or weak PRMT5 expression (Table 2). Statistical analysis revealed that expression levels of PRMT5 significantly increased in cancer tissues compared to the corresponding NATs (Figure 2A-2C). In addition, nuclear but not cytoplasmic PRMT5 level was negatively correlated with the survival rate of CRC patients (Figure 2D). However, no significant association was found between PRMT5 expression in CRC and tumor size, TNM stage, or differentiation (*P* > 0.05, Table 1). Taken together, these results reveal that PRMT5 overexpression is tightly linked to CRC development.

**Table 1:** Clinical and pathological characteristics of patients with colorectal cancer (n = 90)

Characteristics	n	PRMT5 score	*P* value
Gender			
Male	47	4.6011	0.258
Female	43	4.9535	
Age (years)			
≤60	16	4.5468	0.507
>60	74	4.8176	
Tumor size (cm)			
≤4	30	4.5917	0.420
>4	60	4.8704	
TNM stage			
I	9	4.3889	0.259
II	47	5.0106	
III	34	4.5368	

**Table 2:** Summary of PRMT5 composite scoring distribution

Composite score	Normal colorectal tissue (n = 90)	CRC tissue (n = 90)
≤2.0 (weak immunopositivity)	63 (70.0%)	4 (4.4%)
2.1-4.0 (moderate immunopositivity)	17 (18.9%)	30 (33.3%)
>4.0 (strong immunopositivity)	10 (11.1%)	56 (62.2%)
P-values		<0.0001

P-value calculated by χ2 test for Normal colorectal tissue versus CRC tissue.

### AMI-1 significantly inhibits PRMT5 activity

**Figure 3:**
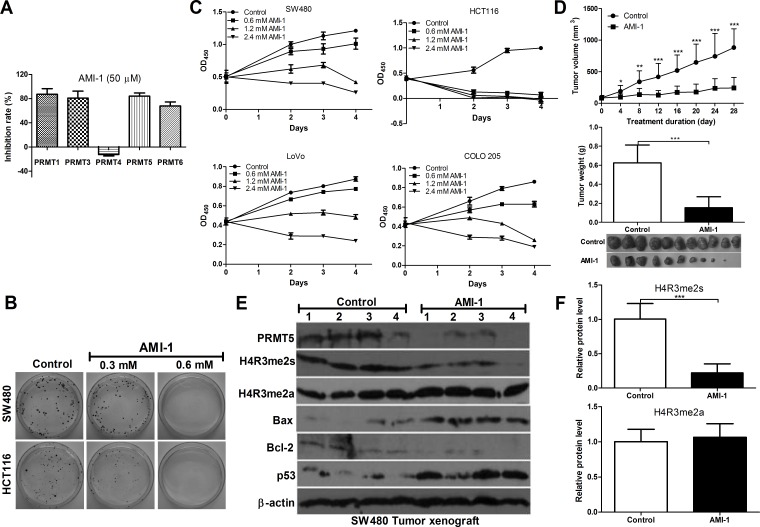
AMI-1 inhibits CRC cell growth *in vitro* and *in vivo* **A.** The inhibitory effects of AMI-1 (50 μM) on PRMTs activity. **B.** The effects of AMI-1 on cell proliferation of human CRC cell lines. **C.** The effects of AMI-1 on colony formation of CRC cell lines. **D.** The effects of AMI-1 on tumor formation in a nude mouse xenograft model. SW480 cells (1 × 10^6^) were injected s.c. into the right flank of nude mice. There were 10-11 mice each in treated and control groups. After 11 d, when tumor nodules were clearly visible, sites were injected 3 times per week with AMI-1 (0.5 mg in 100 μL of vehicle) or 100 μL vehicle (0.9% NaCl). Control animals and animals treated with AMI-1 were euthanized after 28 d and tumor were excised, measured and analyzed. **E.** The expression of pro-, anti-apoptotic protein, H4R3me2s and H4R3me2a in SW480 tumor exnograft was detected by Western blot analysis. **F.** Densitometric analysis of band intensities (H4R3me2s and H4R3me2a) using Image J software (normalized to β-actin). Controls were vehicle-treated group.

Currently, no specific inhibitors of PRMT5 are available for therapy [21]. AMI-1, the first discovered PRMTs inhibitor, is a symmetrical sulfonated urea that inhibits type I enzyme activity *in vitro* [22, 23]. To determine whether AMI-1 inhibits the type II PRMT5 activity, AMI-1 was tested for inhibitory activity against PRMT5 as well as type I enzymes by using PRMTs Chemiluminescent Assay Kit. We found that AMI-1 inhibited 84.2% of PRMT5 activity and also inhibited the activities of PRMT1, 3 and 6. However, PRMT4 activity was not affected (Figure 3A). These results indicate that AMI-1 not only inhibits type I enzymes (PRMT1, 3 and 6), but also inhibits type II PRMT5 activity *in vitro*.

### AMI-1 inhibits CRC cell proliferation *in vitro* and *in vivo*

Next we examined the inhibitory effect of AMI-1 in cultured CRC cells and xenograft CRC mouse models. As shown in Figure 3B and 3C, significant cell growth inhibition was observed in CRC cells exposed to AMI-1 for 48, 72, and 96 h. In CRC animal models, the tumors were injected with AMI-1 intratumorally because AMI-1 via systemic delivery is easily denatured or degraded. AMI-1 markedly reduced tumor volume and weight by 72.5% and 75.5% compared to the control, respectively (Figure 3D).

### AMI-1 inhibits PRMT5 activity in CRC via symmetric dimethylation of histone 4

Because PRMT5-driven methylation of arginine residues leads to symmetric dimethylation of histone 4 (H4R3me2s), we then measured H4R3me2s expression in CRC xenograft nude mice treated with AMI-1. We found that H4R3me2s expression was significantly decreased by AMI-1 compared with the control. To exclude the effect of AIM-1 on type I PRMTs, we measured H4R3me2a level and found no significant change (Figure 3E and 3F). These results suggest that AMI-1 decreases CRC growth, at least in part, through inhibiting PRMT5. In addition, AMI-1 significantly increased Bax/Bcl-2 ratio and p53 levels in tumors (Figure 3E). AMI-1 was also well tolerated, since no significant weight loss in mice was observed (data not shown). These data indicate potent apoptosis activity of AMI-1 against CRC cells *in vivo*.

### AMI-1 induces apoptosis and inhibits migration activity in CRC cells

**Figure 4:**
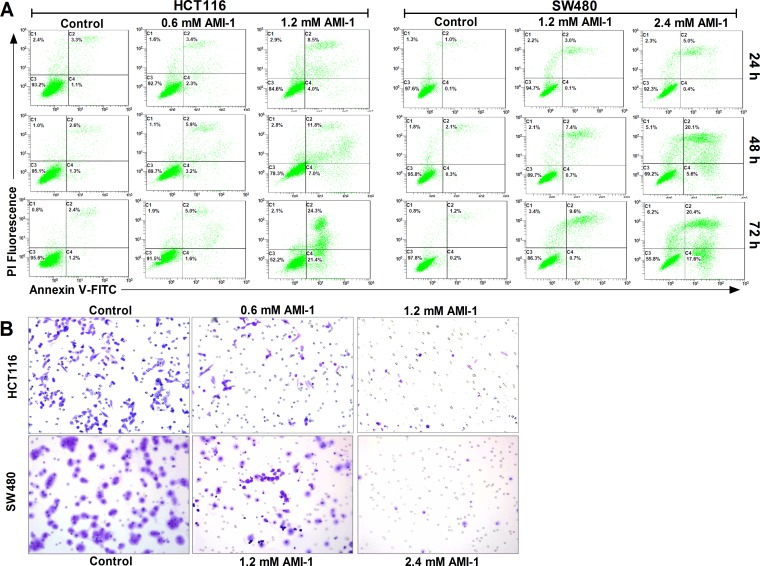
AMI-1 promotes apoptosis and decreases migratory activity of CRC cells **A.** SW480 and HCT116 cells were treated with vehicle alone or AMI-1 and stained by Annexin V-fluorescein isothicyanate (FITC) and propidium iodide (PI), followed by flow cytometry. **B.** AMI-1 decreased migratory activity of CRC cells measured by Transwell assay. Representative photos of stained cells are shown with original magnification 200×. Controls were vehicle-treated group.

PRMT5 silencing can induce apoptosis in different types of cancer [17, 24]. To investigate the effect of AMI-1 on CRC cell apoptosis, two different cell lines (HCT116 and SW480) were treated with either AMI-1 or vehicle only. Flow cytometry analysis showed that AMI-1 resulted in the induction of apoptosis in both cell lines (Figure 4A). We next evaluated the effect of AMI-1 on CRC cell migration, Transwell migration assay showed that treatment of CRC cells with AMI-1 resulted in marked reduction in migration activity compared with control group (Figure 4B).

### Downregulation of PRMT5 suppresses CRC cell proliferation *in vitro* and *in vivo*

**Figure 5:**
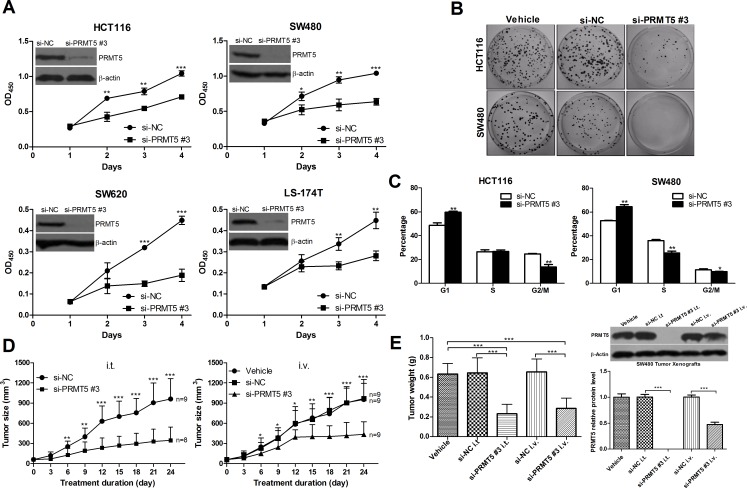
PRMT5 knockdown decreases CRC cell growth *in vitro* and *in vivo* **A.** The effects of si-PRMT5 on cell proliferation of CRC HCT116, SW620, SW480, and LS-174T cells. **B.** The effects of si-PRMT5 on colony formation of CRC cells (HCT116 and SW480). Representative results of colony formation of only vehicle (left), si-NC (middle), and si-PRMT5 (right) SW480 and HCT116 cells. **C.** The effects of si-PRMT5 on CRC cell cycle. SW480 and HCT116 cells were transfected with si-NC or si-PRMT5 and subjected to cell cycle analysis. **D.** and **E.** The effects of si-PRMT5 on tumor formation in nude mouse xenograft model. The tumor volume and weight of si-PRMT5 group was significantly decreased compared with that of control group.

To further corroborate the role of PRMT5 in CRC cell proliferation, three different small interfering RNAs (siRNAs) against human PRMT5 (Supplementary Figure 1A) was used to knockdown PRMT5 in four human CRC cell lines, HCT116, SW480, SW620 and LS-174T. We tested the specificity of PRMT5-specific siRNAs (si-PRMT5) in several CRC cell lines. The results showed that si-PRMT5 did not affect the protein levels of PRMT7 and PRMT1 (Supplementary Figure 1B and 1C). All si-PRMT5 efficiently reduced the levels of endogenous PRMT5 protein (Figure 5, Supplementary Figure 1B and 1C), and significantly inhibited CRC cell proliferation (Figure 5A and Supplementary Figure 3A) and colony formation (Figure 5B and Supplementary Figure 3B). Furthermore, compared with negative control siRNA (si-NC) transfected cells, G1 population of si-PRMT5 transfected cells was significantly increased with a corresponding decrease in S and G2/M phase (Figure 5C and Supplementary Figure 3D). These data indicate that PRMT5 may regulate G1-to-S phase transition.

We next investigated whether si-PRMT5 inhibits the growth of colorectal tumor xenografts in nude mice. On day 7 after xenograt implantation, mice carrying palpable subcutaneous SW480 tumor xenografts received intratumorally (i.t.) or intravenously (i.v.) injections of formulated si-PRMT5. As controls, tumor-bearing animals were injected i.t. or i.v. with formulated si-NC or vehicle alone. Tumors injected with formulated si-NC developed at a pace similar to those that received vehicle. In contrast, injections of formulated si-PRMT5 specifically blocked tumor growth in nude mice (Figure 5D and 5E).

In addition, to assess the safety of systemically delivered si-PRMT5, we examined serum levels of alanine aminotransferase, aspartate aminotransferase, blood urea nitrogen, alkaline phosphatase, creatinine, and creatine kinase from mice treated with formulated si-NC, si-PRMT5 or vehicle alone. As shown in Supplementary Figure 4, the markers indicating the toxicity in the liver, kidney, and heart were within the reference range, suggesting that si-PRMT5 treatment was well tolerated.

### PRMT5 knockdown down-regulates oncogene FGFR3 and eIF4E expression

**Figure 6:**
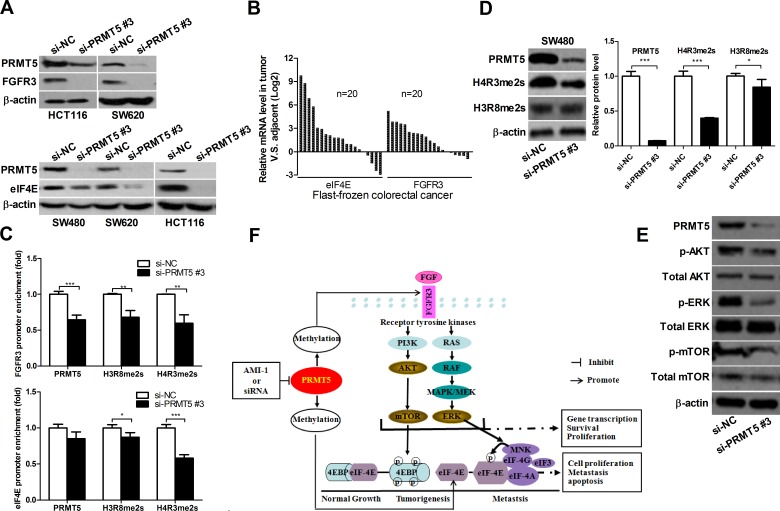
PRMT5 knockdown represses FGFR3 and eIF4E expression and decreases H3R8 and H4R3 methylation on their promoters **A.** HCT116, SW620, and SW480 cells were transfected with si-NC or si-PRMT5. FGFR3 and eIF4E protein were analyzed by immunoblot 72 h later. **B.** FGFR3 and eIF4E mRNA expression in CRC tissues was analyzed by qRT-PCR. **C.** ChIP assays were performed in SW480 cells with antibodies (PRMT5, H3R8, H4R3 and normal IgG), and qRT-PCR with primers targeting control region or part of FGFR3 or eIF4E gene promoter. **D.** SW480 cell were transfected with si-NC or si-PRMT5 and protein expression of H4R3me2s and H3R8me2s was analyzed by Western blot 72 h later. **E.** SW480 cell were transfected with si-NC or si-PRMT5 and the expression of indicated proteins was analyzed by Western blot 72 h later. **F.** Proposed molecular mechanisms by which PRMT5 promotes CRC. PRMT5 overexpression promotes the activation of FGFR3, AKT, mTOR, ERK and eIF4E, leading to CRC cell growth, survival and migration. PRMT5 knockdown or inhibition by AMI-1 results in the downregulation of FGFR3, AKT, mTOR, ERK and eIF4E, leading to the inhibition of CRC cell growth, survival and migration.

It has been reported that FGFR3 and eIF4E play an important role in cell proliferation and promote tumorigenesis [25-27]. PRMT5 exerts its function by regulating the expression of target genes, including oncogene FGFR3 and eIF4E [6, 8, 14]. As shown in Figure 6A and Supplementary Figure 3E, FGFR3 and eIF4E expression were significantly lower in si-PRMT5 CRC cell lines than in si-NC CRC cell lines. To further evaluate the relationship of PRMT5 with eIF4E and FGFR3 in CRC patients, we detected the mRNA levels of FGFR3 and eIF4E in CRC tissue samples. Among 20 cases, FGFR3 and eIF4E expression was markedly elevated in 15 (75%) and 16 (80%) cases of CRC tissues, respectively, compared to the corresponding NATs (Figure 6B). These results suggest that FGFR3 and eIF4E expression are positively correlated with PRMT5 in CRC tissues.

### PRMT5 regulates H3R8 and H4R3 methylation on FGFR3 and eIF4E promoters

Recent studies have shown that the methylation of histones H3 and H4 by PRMT5 plays an important role in the regulation of gene expression [8, 14, 16]. PRMT5 shows a preference toward arginine residue 8 on histone H3 (H3R8) and arginine residue 3 on histone H4 (H4R3) [13]. Therefore, we hypothesized that PRMT5 might regulate FGFR3 and eIF4E expression through the methylation of histones H3 or H4. We performed chromatin immunoprecipitation (ChIP) assay and found that PRMT5 knockdown decreased the binding of PRMT5 to FGFR3 and eIF4E promoters (Figure 6C). In addition, Western blot analysis and ChIP assay using the methyl-histone antibody revealed that PRMT5 knockdown resulted in decreased H3R8 or/and H4R3 methylation in SW480 cell lines (Figure 6C, 6D and Supplementary Figure 3F). Taken together, these findings suggest that PRMT5 binds directly to FGFR3 and eIF4E promoters, and silencing PRMT5 can decrease H3R8 and H4R3 methylation at FGFR3 and eIF4E promoters.

### PRMT5 knockdown suppresses the activation of AKT, ERK and mTOR in CRC cells

FGFR3 is one of the receptors that promote cell survival by stimulating PI3K/AKT/mTOR signaling, and has been shown to activate AKT and ERK in human cancers [25, 26]. To gain further insight into the molecular mechanisms underlying PRMT5-dependent regulation of FGFR3, we examined whether PRMT5 can regulate the activation of AKT, ERK and mTOR through inhibiting FGFR3. As shown in Figure 6E, PRMT5 knockdown significantly decreased the levels of phosphorylated AKT, ERK and mTOR in SW480 cells. These results indicate that PRMT5 promotes the activation of PI3K/AKT/mTOR and ERK signaling in CRC cells.

## DISCUSSION

Knowledge about the precise molecular mechanisms underlying colorectal tumorigenesis is crucial to the development of better therapy strategy for CRC patients. The dysregulation of PRMTs has been observed in diverse types of cancer and the modulation of PRMTs levels affects cancer cell growth, thus PRMTs become promising targets for therapeutic strategies [28]. Here, we focused on PRMT5, which has been reported to be upregulated in lung cancer, leukemia, lymphoma, ovarian cancer, and breast cancer [6, 10, 13, 14, 16, 29]. However, the function and molecular mechanism of histone arginine methylation in oncogenesis is not well understood. In this study, we show that PRMT5 is frequently highly expressed in human CRC tissues and cell lines. The correlation between PRMT5 overexpression and poor clinical outcome of patients with CRC are intriguing with regard to potential utility as a prognostic factor to identify patients with more aggressive disease. Silencing PRMT5 expression significantly inhibits the growth of CRC both *in vivo* and *in vitro*. Thus, it appears that PRMT5 is a key histone-modifying enzyme that controls cell growth by modulating the expression of target genes through histone arginine methylation.

Arginine methylation is a very stable mark because it is unclear if the modification can be enzymatically reversed. Two types of putative arginine demethylases have been reported: Jumonji domain-containing protein (JMJD6) and peptidylarginine deiminases (PADIs) [30, 31]. However, they are not “true” demethylases because they can not remove the methyl-group from methylated arginine residues and free arginine from the derivatives. Recently, Webby et al. reported that JMJD6 is actually a lysine hydroxylase and structure analysis of JMJD6 suggests that it is not an arginine demethylase [32]. Therefore, arginine methylation probably represents a “long term” activating mark in gene transcriptional regulation. Considering persistent nature of cancer, we assume that modified binding of H4R3me2s and H3R8me2s at the promoters of eIF4E and FGFR3 may persistently influence oncogene expression in CRC and maintain oncogene-induced tumorigenicity.

Numerous efforts are currently underway to develop PRMTs inhibitors as chemical probes and therapeutic reagents [33, 34]. PRMTs are evolutionarily conserved from the yeast to human and commonly classified as type I and II based on the nature of methylation [35]. Type I PRMTs (PRMT1, 2, 3, 4, 6 and 8) catalyze the production of asymmetrically dimethylated, whereas type II PRMTs such as PRMT5 catalyze the formation of symmetrically dimethylated [21, 35]. In this study, we demonstrate that AMI-1 is able to inhibit PRMT5 activity in human CRC tumor tissues. Although AMI-1 also inhibits the activities of PRMT1, 3 and 6 *in vitro*, our results revealed the *in vivo* substrate selectivity of AMI-1, since no significant change was observed in the expression of H4R3me2a, a histone mark of type I PRMTs. These findings are not surprising, because PRMT5 is markedly upregulated in CRC. In addition, it has been reported that PRMT3 and PRMT6 are mainly overexpressed in breast, bladder and lung cancer but not CRC [21]. These results indicate that AMI-1 decreases CRC growth, at least in part, through inhibiting PRMT5.

Because PRMT5 has been shown to mediate arginine methylation of p53 to regulate its function [11, 17], we explored the effects of AMI-1 on p53 in nude mouse CRC xenograft model. Interestingly, we found that PRMT5 is required for p53 expression and PRMT5 inhibition by AMI-1 prevents p53 protein synthesis along with a significantly elevated Bax/Bcl-2 ratio. FGFR3 is one of the receptors that promote cell survival by stimulating PI3K-AKT signaling [36]. FGFR3 is frequently overexpressed in myeloma, ovarian and bladder cancers, suggesting its role in tumorigenesis [37, 38]. eIF4E binds the 5′,7-methylguanosine cap structure of mRNAs, delivering these mRNAs to eIF4F translation initiation complex composed of eIF4E, scaffolding protein eIF4G, and ATP-dependent RNA helicase eIF4A. The eIF4F complex then scans through the 5′ untranslated region (UTR), unwinding mRNA secondary structure to expose the translation initiation codon and enable translation. The eIF4E complex assembly is rate limiting for the translation and is largely dependent on eIF4E availability [39, 40]. Moreover, eIF4E is a key event downstream of *ras* and phosphatidylinositol 3-kinase/protein kinase B/mammalian target of rapamycin (PI3K/AKT/mTOR) signaling pathway, which are frequently activated in a diverse range of human cancer [41, 42]. Therefore, in the present study inhibiting FGFR3 or eIF4E selectively suppressed the expression of FGFR3 or eIF4E regulated proteins and substantially repressed tumor growth on tumor models.

Both FGFR3 and eIF4E have been shown to promote tumor growth, and are overexpressed in human cancers including CRC [37-39, 41, 43]. However, the mechanism and role of FGFR3 and eIF4E in CRC has not yet been elucidated. Consistent with previous reports, we showed that FGFR3 and eIF4E were significantly upregulated in human CRC samples. Our ChIP results demonstrated that PRMT5 was recruited to FGFR3 or eIF4E-binding sites within the promoter regions of these target genes. We assume that PRMT5 catalyzes the histone methylation at these promoter regions, enhancing the transcription of these genes. Interestingly, we found that PRMT5 plays a profound role in the FGFR3 pathway and participates in regulating FGFR3 downstream targets such as AKT, ERK and mTOR. Thus, silencing PRMT5 in CRC cells reduces FGFR3 expression, leading to the repression of AKT and ERK and subsequent inhibition of mTOR through the AKT/mTOR or ERK pathway. On the basis of these findings, we propose a model for the function of PRMT5 specific siRNA or inhibitor AMI-1 in tumor growth (Figure 6F). We provide the first line of evidence to show that FGFR3 and eIF4E overexpression in CRC may result from PRMT5 overexpression, indicting a new regulatory mechanism for arginine methylation to drive CRC cell proliferation. This finding also suggests that PRMT5 mediated methylation is bi-functional, either repressing or promoting transcription, entirely depending on which genes are regulated [44]. How PRMT5 have both activation and repression capacity in cell proliferation and differentiation is unclear. It is possible that PRMT5 affects other repressive/activator chromatin remodeling complexes to regulate gene transcription. It was reported that PRMT5 interacts with BRG1 and hBRM based hSWI/SNF chromatin remodelers and both complexes specifically methylate histones H3 and H4 [10]. Considerable additional work will be required to distinguish between the multiple potential mechanisms for PRMT5 function in different gene regulation events.

PI3K/AKT/mTOR signaling regulates several cellular functions that are critical to tumorigenesis such as cellular proliferation, growth, survival and mobility [45]. A major downstream target of PI3K/AKT/mTOR pathway is eukaryotic translation initiation factor 4E binding protein 1 (4EBP1), which plays a crucial role in the regulation of protein synthesis [46]. It has been observed that mTOR is often overexpressed and activated in a wide variety of human cancers but the underlying mechanisms remains unclear [47, 48]. This model (Figure 6F) for PRMT5 function in CRC cells would provide a missing link between FGFR3, mTOR, 4EBP1, eIF4E and cancer progression.

Taken together, our results show that PRMT5 is frequently overexpressed in human CRC tissues and cell lines. PRMT5 knockdown or PRMT5 inhibitor significantly inhibits cell proliferation *in vitro* and *in vivo*. PRMT5 overexpression represents an unfavorable prognostic marker and an attractive therapeutic target for CRC. Expanding insight into the dysregulation of oncogenes through epigenetic mechanism in colorectal tumorigenesis will yield important clues to improve our understanding of the complicated molecular pathogenesis of CRC and facilitate the development of new therapeutic regimens for CRC patients.

## MATERIALS AND METHODS

### Human tissue samples and cell lines

Forty eight flash-frozen CRC tumor samples and corresponding normal adjacent tissues (NATs) were collected between 2011 and 2012 at State Key Laboratory of Biotherapy and Cancer Center, West China Hospital, Sichuan University. Tissue samples were immediately snap-frozen in liquid nitrogen. Ninety pairs of CRC tissues and adjacent NATs arrays were purchased from the National Engineering Center for Biochips (Shanghai, China). Both tumor and adjacent NAT were histologically examined. All human materials were obtained with informed consent, and this project was approved by Ethics Committee of Sichuan University. Seven human CRC cell lines, including HCT116, SW480, CX-1, SW620, LoVo, COLO 205, LS-174T and normal colonic mucosal FHC cells, were purchased from American Type Culture Collection (ATCC; Manassas, VA, USA), and cultured under conditions recommended by ATCC.

### RNA extraction and quantitative real-time PCR

Total RNA from CRC tumor samples was extracted using TRIzol reagent (Invitrogen, Carlsbad, CA, USA). cDNA was synthesized with the iScript™ cDNA Synthesis Kit (Bio-Rad, Hercules, CA, USA). Quantitative real-time PCR (qRT-PCR) analyses were carried out to detect mRNA expression using iQ™ SYBR^®^ Green Supermix (Bio-Rad), and β-actin gene was used as an internal control. PRMT5, FGFR3, and eIF4E expression were determined by using qRT-PCR primers according to the provided protocol. Raw CT values were normalized to the values of β-actin to correct for equal input of RNA, and analysis of gene expressions was performed using the 2^−ΔΔCt^ method. The qRT-PCR primers are listed in Supplementary Table 1.

### Tissue microarray and evaluation of immunohistochemical staining

Tissue arrays were constructed using 90 pairs of CRC tissues and adjacent NATs. Immunohistochemical staining was performed on 4 μm sections of paraffin-embedded human CRC tissues and matched NATs. Briefly, the slides were incubated in 200 μg/mL PRMT5 antibody (1:600; Santa Cruz Biotech, Santa Cruz, CA, USA) overnight at 4°C. Subsequent steps were performed using Polymer Detection System for Immuno-Histological Staining (ZSGB-BIO, PV-9000, Beijing, China) according to the manufacturer's instruction. PRMT5 expression in the tissues was evaluated by immunohistochemical staining with a PRMT5-specific antibody. A semi-quantitative scoring method according to the overall staining of the cells was applied for IHC [49, 50]. The intensity of protein expression was graded as follows: 0, no staining; 1, weak; 2, moderate; 3, strong. The staining density was quantified as the percentage of cells staining positively as follows: 0, no staining; 1, 1-25% of cells stained; 2, 26-50% of cells stained; 3; 51-75% of cells stained; and 4, >75% of cells stained. Intensity score was multiplied with density score to yield an overall score of 0-12 for each specimen. Each slide was read and scored independently by two pathologists in a blinded fashion.

### PRMTs enzymatic assay

PRMTs enzymatic assays were conducted using commercial PRMTs Chemiluminescent Assay Kit (BPS Bisciences, CA, USA) following the protocols. Briefly, histones were coated in a high binding plate and incubated with histone methyltransferase (HMT) assay buffer, S-adenoslymethionine, PRMTs enzyme, and AMI-1 (test inhibitor, 50 μM). Then, primary antibody was added. Finally, the plate was treated with an HRP-labeled secondary antibody followed by the addition of HRP substrate to produce chemiluminscence that can then be measured using a BioTek Synergy™ 2 Multi-mode Microplate Reader.

### Colorectal cancer xenografts

Male athymic BALB/c nude mice were purchased from Vital River Laboratory Animal Technology Co. Ltd. (Beijing, China) and maintained under specific pathogen-free conditions in the National Chengdu Center for Safety Evaluation of Drugs. Mice were manipulated and housed according to protocols approved by the Institutional Animal Care and Treatment Committee of Sichuan University. A total of 1.0× 10^6^ SW480 cells were inoculated in 0.1 mL of serum-free medium subcutaneously in the right flank of 5–6-week old healthy BALB/c nude mice. When the tumors reached an average volume of about 60 mm^3^, the mice bearing too large or too small tumors were eliminated and the left were divided into groups for treatment. Turing the experiment, tumor size was measured using with calipers every three days, and tumor volumes were calculated according to the following formula: (*Length* × *Width*^2^) × 0.52.

### siRNA delivery

Three different siRNAs for human PRMT5 were provided by Ribobio (Guangzhou, China) and their sequences are listed in Supplementary Figure 1A. Cells were transiently transfected with a PRMT5 siRNA duplex (si-PRMT5; final concentration of 50-100 nmol/L) or a control siRNA (si-NC, a random scrambled sequence) using Lipofectamine 2000 (Invitrogen, Carlsbad, CA, USA) according to the manufacturer's instructions. Following transfection, cells were subjected to growth inhibition, flow cytometric, and immunoblot analyses. For *in vivo* delivery, PRMT5 siRNAs and its negative control (Cat. No. siN05815122147; Guangzhou, China) were conjugated with cholesterol and were administered either intratumorally (i.t.) or intravenously (1 nmol oligo/each mouse, twice a week ).

### Cell proliferation assay, colony formation assay cell cycle and apoptosis analysis

To determine cell growth rate, 2.5 × 10^3^ cells were seeded per 96–well plate. After 24 h, cells were transfected with si-NC or si-PRMT5. At times indicated, proliferation assay was performed with the Cell Counting Kit-8 (CCK8; Dojindo). For the colony formation assay, cells were placed in 60 mm dishes at 200 cells per dish and maintained in the absence or presence of PRMT5 siRNA for 14 days. Cells were fixed with fixative (7 parts methanol: 1 part glacial acetic acid) for 15 min and then stained with crystal violet (0.2 g/liter) for 25 min. For cell cycle assay, cells were plated at 1 × 10^5^ cells/well in 6-well plates. The next day, cells were treated with AMI-1 or vehicle control only for 24 h, 48 h and 72 h. The treated cells were harvested using 0.25% trypsin without EDTA and fixed in 75% ethyl alcohol. After digestion with RNase, DNA was stained with propidium iodide and analysis with a Beckman Coulter flow cytometer and Modfit software. For apoptosis assay, treated cells were stained with Annexin V and propidium iodide and evaluated using a Beckman flow cytometer.

### Migration assay

Assays were conducted using 8 μm pore size Transwell plate (Corning Costar). In brief, approximate 4 × 10^4^ cells resuspended in 100 μL non-serum culture medium were placed triplicatedly in upper chamber of insert and medium with 10% FBS was used as chemo-attractant in lower chamber. AMI-1 or vehicle was added to inner chamber. Before plating in the outer chamber, cells were incubated for 20 min with RPMI 1640 medium containing AMI-1 or vehicle. Cells were allowed to migrate for 20 h. The cells remaining on the top surface of the membrane were removed with a cotton swab. The cells on the bottom surface of the membrane were fixed and stained with 0.2% crystal violet.

### Immunoblot analysis

Protein were separated in 6%-12% SDS-PAGE gels and then transferred to PVDF membrane (Bio-Rad). After blocking with 5% non-fat milk for 1 h, the membranes were incubated with the primary antibodies at 4°C overnight. After washing with 1 × TBST, the membranes were incubated with corresponding secondary antibodies conjugated to horseradish peroxidase (ZSGB-BIO, Beijing, China). Immunoreactivity was visualized using ECL Western blotting detection reagents and then analyzed through scanning densitometry. The identity and suppliers of the antibodies are listed in Supplementary Table 2.

### Chromatin immunoprecipitation (ChIP) assay

ChIP was performed with SW480 cells transfected with si-NC or si-PRMT5 using EZ-Magna ChIP G kit (Millipore, Billerica, MA, USA) according to the manufacturer's protocol. Protein-DNA complexes were precipitated with normal IgG, anti-PRMT5, anti-Histone H3 (Sym-dimethyl Arg8) and anti-Histone H4 (Sym-dimethyl R3) antibodies at 4°C overnight with rotation. The abundance of FGFR3, GAPDH, and eIF4E promoter regions in ChIP precipitates was quantified using qRT-PCR and specific primers. The qRT-PCR primers are listed in Supplementary Table 1, and the antibodies used in Supplementary Table 2.

### Statistical analysis

The results were presented as mean ± S.D., except where indicated. The data were subjected to student's *t* test. *P*-value less than 0.05 was considered statistically significant. Error bars, S.D. *P < 0.05, **P < 0.01 or ***P < 0.001 level.

## SUPPLEMENTARY MATERIAL FIGURES AND TABLES


