# Supplementary material for: Targeting protein arginine methyltransferase 5 inhibits colorectal cancer growth by decreasing arginine methylation of eIF4E and FGFR3
Source: Oncotarget. 2015 Jun 2;6(26):22799–811. doi: 10.18632/oncotarget.4332 (PMC4673200; doi:10.18632/oncotarget.4332)
Supplement: Supplementary file 1 [file oncotarget-06-22799-s001.pdf]

Targeting protein arginine methyltransferase 5 inhibits colorectal cancer growth by decreasing arginine methylation of eIF4E and FGFR3

SupplementaryMaterial

A

No:	si-PRMT5 #1
Target sequence	GACCTGAGAGATGATATA
Sense strand	5′ GGACCUGAGAGAUGAUAUA dTdT 3 ′
Antisense strand	3′dTdT CCUGGACUCUCUACUAUAU 5 ′
No:	si-PRMT5 #2
Target sequence	CCAGAAGAGGAGAAGGATA
sense strand	5′ CCAGAAGAGGAGAAGGAUA dTdT 3 ′
antisense strand	3′ dTdT GGUCUUCUCCUCUUCCUAU 5 ′
No:	si-PRMT5 #3
Target sequence	CCGCTATTGCACCTTGGAA
Sense strand	5′ CCGCUAUUGCACCUUGGAA dTdT 3 ′
Antisense strand	3′ dTdT GGCGAUAACGUGGAACCUU 5 ′

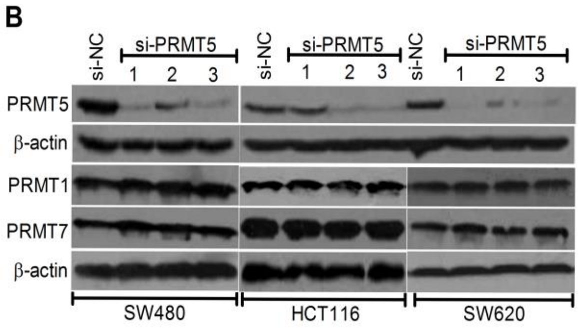

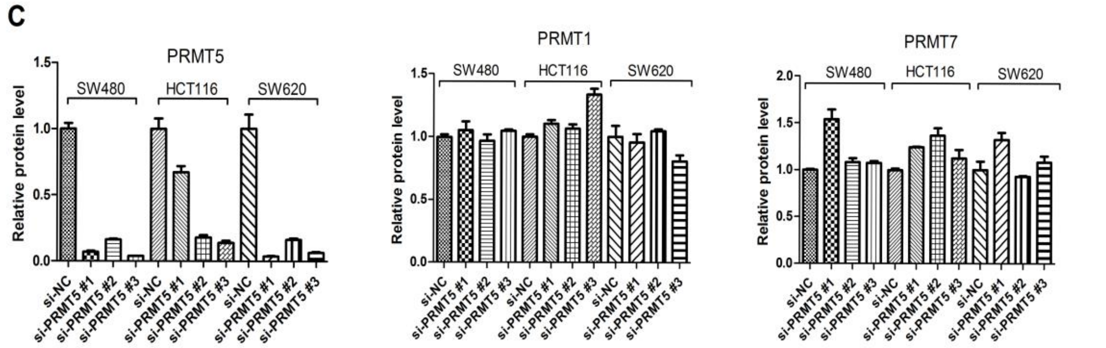

Supplementary Figure 1: The PRMT5 siRNA sequences and their effects on PRMT5, PRMT1 and PRMT7 expression in CRC cells.

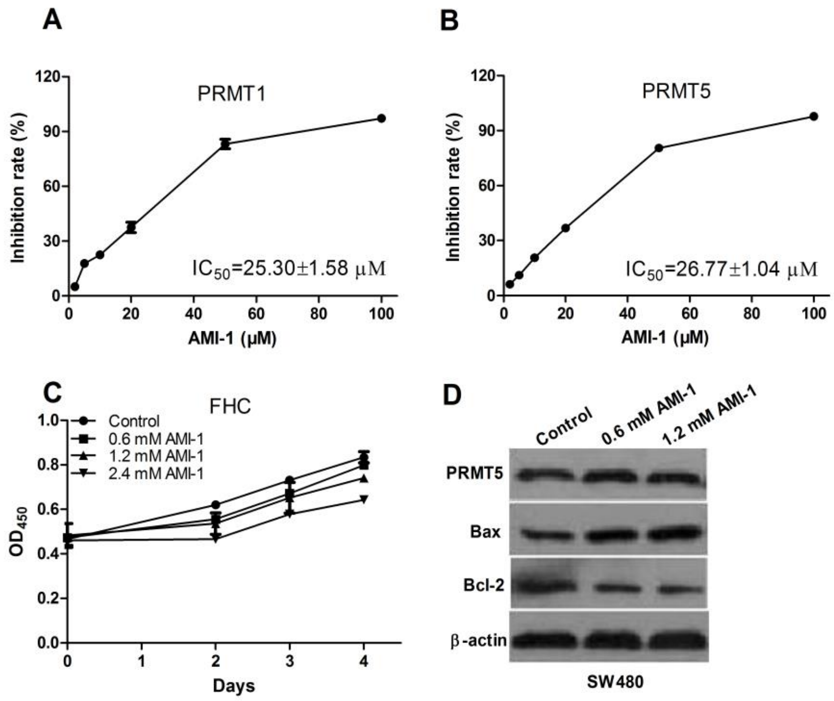

Supplementary Figure 2: (A) The effect of AMI-1 on PRMT1 activity. **(B)** The effect of AMI-1 on PRMT5 activity. **(C)** The effect of AMI-1 on the proliferation of normal colonic mucosal FHC cells. **(D)** SW480 cells were treated with AMI-1 for 48 h. Protein expression of Bax and Bcl-2 were analyzed by Western blot analysis.

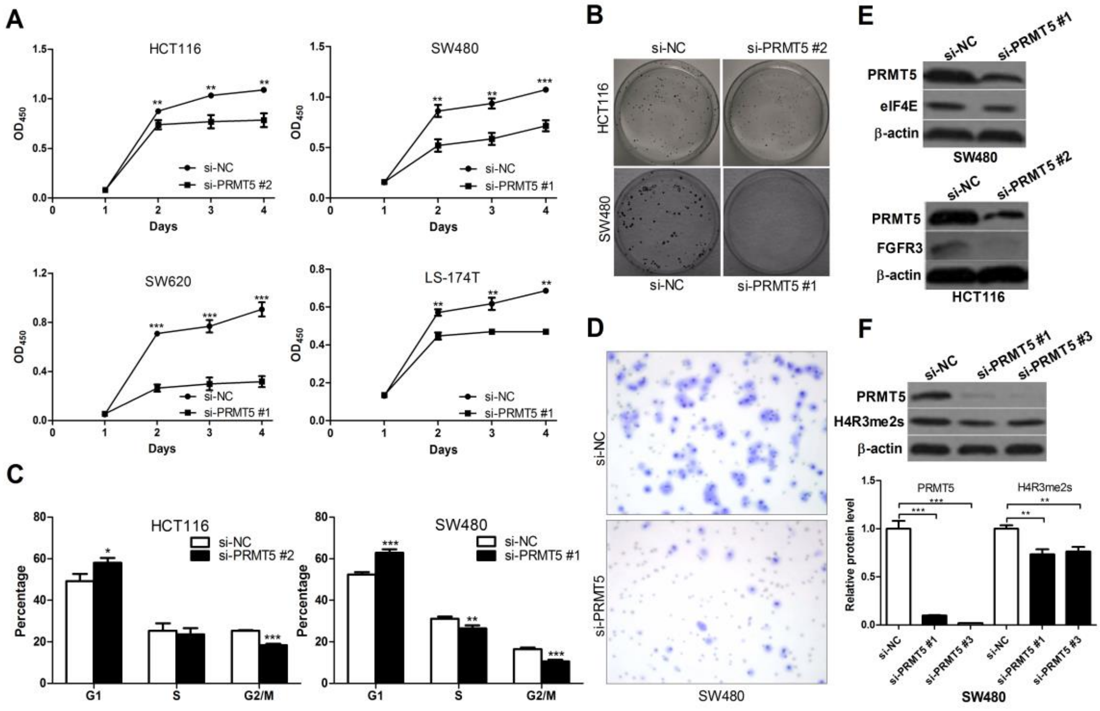

Supplementary Figure 3: Silencing PRMT5 decreases CRC cell growth in *vitro*. (A) The effects of si-PRMT5 on the proliferation of CRC HCT116, SW620, SW480, and LS-174T cells. **(B)** The effects of si-PRMT5 on colony formation of CRC cells (HCT116 and SW480). **(C)** Knockdown of PRMT5 led to cell G1 arrest. **(D)** Knockdown of PRMT5 decreased migratory activity of CRC cells measured by Transwell assay. Representative photos of stained cells are shown with original magnification 200×. Controls were vehicle-treated group. **(E)** SW480 and HCT116 cells were transfected with si-NC or si-PRMT5 and FGFR3 and eIF4E protein expression was analyzed by Western blot analysis 72 h later. **(F)** SW480 cells were transfected with si-NC or si-PRMT5 and H4R3me2s level was analyzed by Western blot analysis 72 h later.

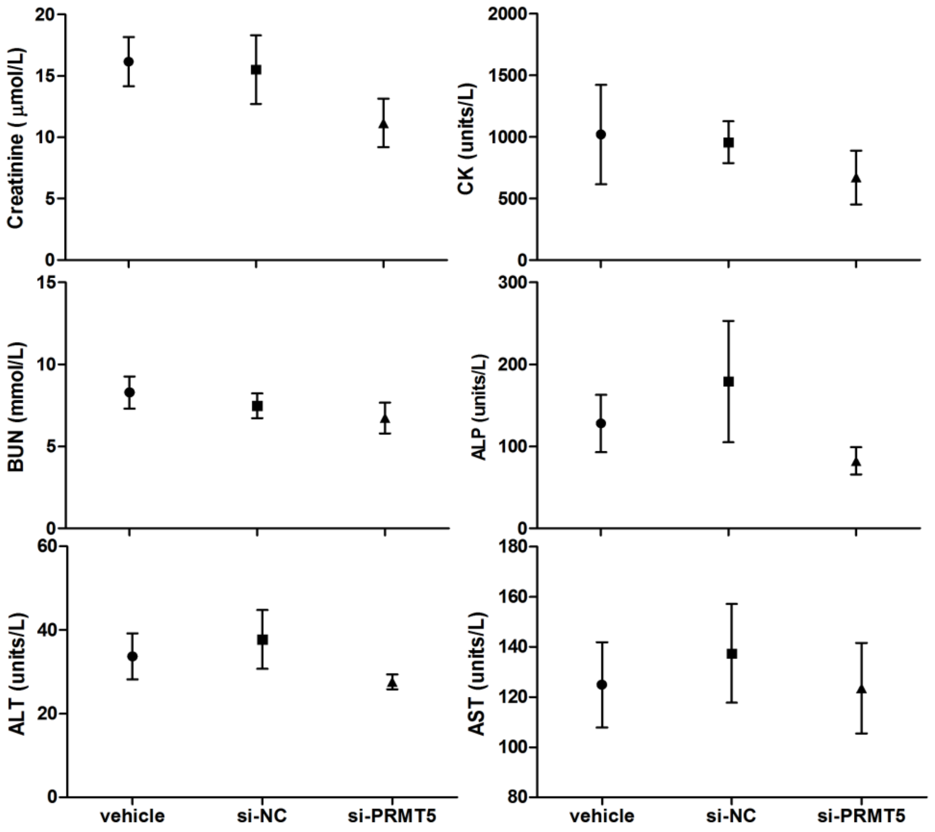

Supplementary Figure 4: Serum levels of blood urea nitrogen (BUN), creatinine, alkaline phosphatase (ALP), alanine aminotransferase (ALT), aspartate aminotransferase (AST), and creatine kinase (CK) of the animals described in Fig. 5E after sacrifice. Points, average of five animals**.**

Supplementary Table 1: Primers used in this study

Method	Primer name	Sequence
qRT-PCR	β-actin-F	5'-TGACGTGGACATCCGCAAAG-3'
	β-actin-R	5'-CTGGAAGGTGGACAGCGAGG-3'
	PRMT5-F	5'-CAATGAGGTCCGAGCCTGTAG-3'
	PRMT5-R	5'-GCGGTTGTTGTCAATCATAGGA-3'
	FGFR3-F	5'-AGGACGAGGCTGAGGACACA-3'
	FGFR3-R	5'-TGGAGGGAGTGGGGTTGC-3'
	eIF4E -F	5'-GAAAACGGAATCTAATCAGGAGG-3'
	eIF4E -R	5'-GCCCAAAAGTCTTCAACAGTATCA-3'
qPCR-ChIP	Primer name	Sequence
	GAPDH-F	5′-TACTAGCGGTTTTACGGGCG-3′
	GAPDH-R	5′-TCGAACAGGAGGAGCAGAGAGCGA-3′
	FGFR3-F	5'-ATGGTGAAGCCCCGTCTCTAC-3'
	FGFR3-R	5'-CTGCCTCCCTGATTGAAGTGA-3'
	eIF4E -F	5'-TGGTAGAATCTCAAATAGGCAAAG-3'
	eIF4E -R	5'-ACAGAGTTTCAGGAAAATCTCCA-3'

Supplementary Table 2: Antibodies used in this study

Antibodies	Company	Catalog Number	Western blot	ChIP
PRMT5(A-11)	Santa Cruz Biotech, Santa Cruz, CA, USA	sc-376937	1:400	2 µg
eIF4E(P-2)	Santa Cruz Biotech	sc-9976	1:500	
FGFR3(C-15)	Santa Cruz Biotech	sc-123	1:200	
Bax(N-20)	Santa Cruz Biotech	sc-493	1:200	
Bcl-2(N-19)	Santa Cruz Biotech	sc-492	1:500	
β-Actin(13E5)	Cell Signaling, Danvers, MA, USA	4970	1:2000	
Phospho-Akt (Thr308)(244F9)	Cell Signaling	4056	1:1000	
PRMT7 (D1K6R)	Cell Signaling	4762	1:1000	
AKT(pan)(11E7)	Cell Signaling	4685	1:1000	
Phospho-p44/42 MAPK (Erk1/2) (Thr202/Tyr204) (D13.14.4E) XP	Cell Signaling	4370	1:2000	
p44/42 MAPK(Erk1/2)(137F5)	Cell Signaling	4695	1:2000	
Phospho-mTOR (Ser2448) (D9C2)XP	Cell Signaling	5536	1:1000	
mTOR(7C10)	Cell Signaling	2983	1:1000	
p53 antibody	Cell Signaling	9282	1:1000	
H3(Me2)R8	NOVUS	NB21-1063	1:250	4 µg
H4(Me2)R3	Abcam	Ab5823	1:2000	2 µg
PRMT1	Abcam	Ab7027	1:800	
IgG	Millipore			1 µg
